# Hierarchical elimination selection method of dendritic river network generalization

**DOI:** 10.1371/journal.pone.0208101

**Published:** 2018-12-03

**Authors:** Chengming Li, Wei Wu, Yong Yin

**Affiliations:** 1 Chinese Academy of Surveying and Mapping, Beijing, China; 2 College of Geomatics, Shandong University of Science and Technology, Qingdao, China; Universidade Federal de Uberlandia, BRAZIL

## Abstract

Dendritic river networks are fundamental elements in cartography, and the generalization of these river networks directly influences the quality of cartographic generalization. Automatic selection is a difficult and important process for river generalization that requires the consideration of semantic, geometric, topological, and structural characteristics. However, owing to a lack of effective use of river features, most existing methods lose important spatial distribution characteristics of rivers, thus affecting the selection result. Therefore, a hierarchical elimination selection method of dendritic river networks is proposed that consists of three steps. First, a directed topology tree (DTT) is investigated to realize the organization of river data and the intelligent identification of river structures. Second, based on the “180° hypothesis” and “acute angle hypothesis”, each river is traced in the upstream direction from its estuary to create the stroke connections of dendritic river networks based on a consideration of the river semantics, length, and angle features, and the hierarchical relationships of a dendritic river network are then determined. Finally, by determining the total number of selected rivers, a hierarchical elimination algorithm that accounts for density differences is proposed. The reliability of the proposed method was verified using sample data tests, and the rationality and validity of the method were demonstrated in experiments using actual data.

## Introduction

Cartographic generalization is a process for recognizing the spatial environment [1]. Franz [2] defined this process as the “selection and simplified representation of detail appropriate to the scale and/or purpose of the map.” In a geographic information system (GIS), generalization can also be considered as a computational procedure for presenting and abstracting spatial data [3]. River networks describe the interconnectivity and distribution of natural rivers and represent a fundamental component of geoinformatics as well as an indispensable skeleton in topographic representations. Two issues are observed with the process of automatic river network generalization: river selection and graphic generalization [4]. This paper focuses on the problem of automatic river selection.

River networks are often manifested in the form of dendritic, anastomosed, or feathery structures. The generalization of anastomosed rivers can use road network selection methods because of similar structural characteristics; therefore, this selection method is well established. Dendritic river networks have clear hierarchical structures and density characteristics. Specifically, the main river and its tributaries exhibit “parent-child” spatial relationships and do not present closed loops, and the river network densities tend to vary from one region to another. Map generalization is scale dependent. The square-root-type model proposed by Topfer [5] provides a relatively simple solution to the question of how many rivers should be selected for a given scale change. However, identifying the rivers that should be deleted or retained is an entirely different issue, and the objective of the cartographer is always to obtain the optimal automatic river selection [6].

The earliest method [7, 8] of river selection was a simple selection model in which researchers selected the river by using river lengths and the number of rivers per unit area. Although this method is easy to implement, the river selection results are not reasonable. Ai [9] pointed out that for river network generalization, we must consider spatial distribution patterns and the distribution density. Many researchers have examined structured selection models of river networks, such as fuzzy mathematical, graph theory, and hierarchical relationship methods, which are detailed as follows.

- The fuzzy mathematical method: This method [10] incorporates four factors in river selection, river length, river density, relative importance, and river network type, which are used as the evaluation set for river selection. A fuzzy comprehensive evaluation matrix is created to perform the river selection. The fuzzy mathematical method accounts for the spatial distribution of the rivers and can determine which rivers should be selected. However, this method is highly reliant on subjective and empirical judgments, which increases the difficulty of automatic selection.
- Graph theory method: River networks on topographic maps organized by river segment can be studied in terms of the "tree" in graph theory [11]. The mainstream and tributaries are determined according to the length and angle of the river segment and the degree of the river node [12], where angle recognition is based on two important hypotheses proposed by Paiva *et al*. [13], the “180° hypothesis” and “acute angle hypothesis”. In the selection process, the mainstreams are retained and tributaries are deleted. The graph theory method makes full use of the geometric characteristics of the river but does not consider the semantic and structural characteristics of rivers. In addition, determining the selection criteria is difficult.
- Hierarchical relationship method: Many researchers have examined this method to construct hierarchical relationships for river networks. Horton [14] first developed the Horton stream-ordering method, and Strahler [15] advanced Horton’s theory to propose the Strahler stream-ordering method. These two methods have been found to closely approximate generalized decisions made by human cartographers [16]. Stanislawski [17] proposed a stratified pruning (SP) method of hydrographic networks on the basis of Strahler stream-ordering. The SP method is good at retaining density variations that depict natural terrain differences [18], and it was being tailored for different terrain (mountains, hills and flat) and climate (humid and dry) conditions within United States by Stanislawski *et al*. [19–21]. However, the SP method does not account for the effects of human perceptual grouping principles. Thomson *et al*. [22, 23] proposed a method for connecting river strokes based on the Gestalt principles [24–26] of “good continuation”. Savino *et al*. [27] put forward a length and density pruning (LaDP) method by using river strokes, the strokes were constructed based on enriched geometric values and available attributes for feature type and name. The LaDP method is tailored to maintain the full extent of river courses.

Currently, the selection of rivers via the hierarchical relationships between river flows is usually performed using a “top to bottom” (from level 1 to *n*) approach [28]. In other words, the mainstreams of the river networks are preserved in a level-by-level approach during the river selection process. Although this approach retains the mainstreams that are more important in the river networks, certain tributary basins are often entirely deleted, thus distorting the spatial distribution and morphology of the rivers. In addition, the connectivity of the river cannot be guaranteed at the edges of the river networks, which could result in cut-off rivers.

To address these issues, the semantic, geometric, and topological characteristics of river networks are combined with the Gestalt principles of perception to construct hierarchical relationships for dendritic river networks in this paper. Subsequently, the structural characteristics of river networks, such as river spacing and network density, are determined, and a river selection method via hierarchical elimination is proposed to perform the automated selection.

## Materials and methods

### Process diagram for the hierarchical elimination selection of a dendritic river network

**Fig 1:**
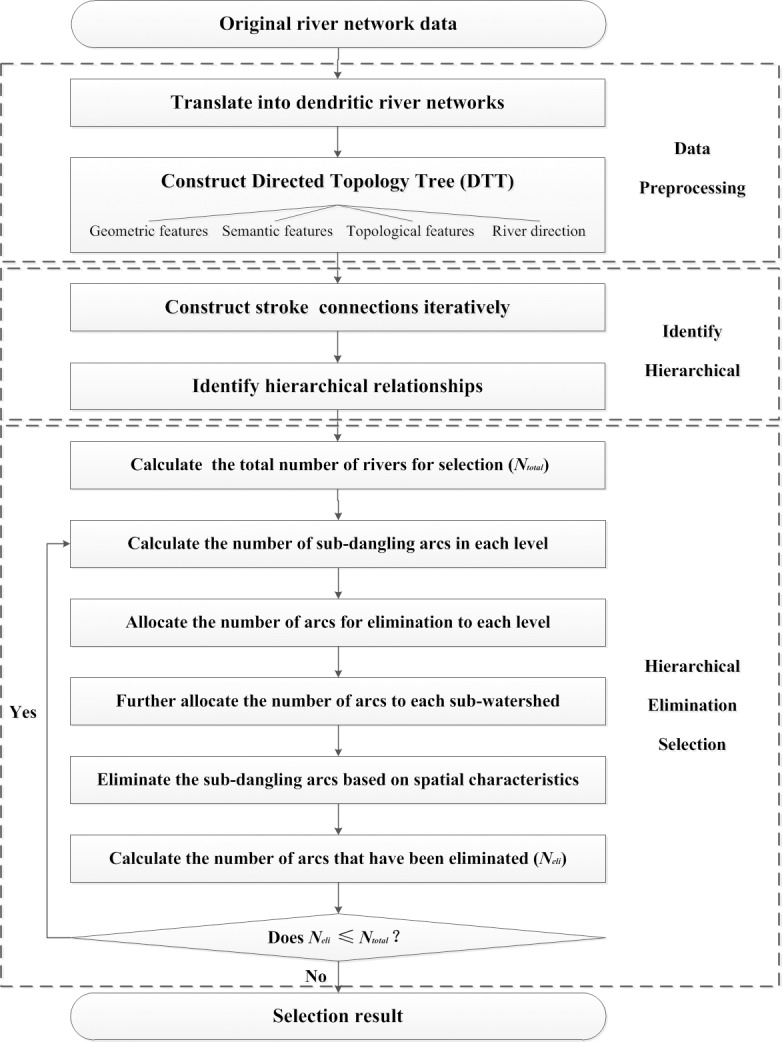
The process diagram for the proposed method.

Our proposed method of automated hierarchical elimination selection for dendritic river network generalization is composed of three main steps (Fig 1) as explained below.

1. Date preprocessing
  Generally the original river network data is complicated, we need to translate the original river network data into basic dendritic river network, then construct Directed topology tree (DTT) based on the geometric, semantic, topological and direction features of the rivers to organize these data.
2. Identify hierarchical
  Firstly the stroke connections were constructed iteratively for each river arc via the comprehensive use of river features, then the hierarchical relationship of the dendritic river network can be identified based on the stroke connections.
3. Hierarchical elimination selection
  Using the square-root-type model to calculate the total number of rivers for selection, then select rivers via hierarchical elimination from outside to inside to obtain the final generalization result.

### Directed topology tree (DTT)

River networks can be manifested in complex and diverse forms on maps. However, most river networks exhibit dendritic shapes. In other words, river networks have mainstreams and tributaries that are related by distinct hierarchical relationships. Scholars have proposed structured mathematical models for river networks based on river arcs. These models describe complex river networks according to the principles of graph theory and topology, thus realizing a unified representation for actual rivers. In this study, complex river networks were divided into topological arcs. A directed topology tree was then used to perform the structured organization of river networks based on previously described principles.

Topological diagrams of dendritic river networks that contain flow directions are called DTTs, which are collections of nodes and arcs that contain information about total flux, inflows, outflows, etc. The direction of an arc is defined as the direction of flow from the starting node to the ending node. In the spatial databases of actual maps, a river is divided into several arcs due to its confluences with other rivers. Nonetheless, the semantic (map layers, elements, names, etc.) and geometric (lengths, angles, flow directions, etc.) river information will be incorporated in its arcs. For example, the element name (featureID), river name (nameID), and layer name (layerID) of an arc represent the semantic attributes of the river. The geometry of the arc includes information on the river’s geometric shape, such as its angles, length, etc. Then a DTT is constructed from the geometric, semantic and topological features of a river.

**Fig 2:**
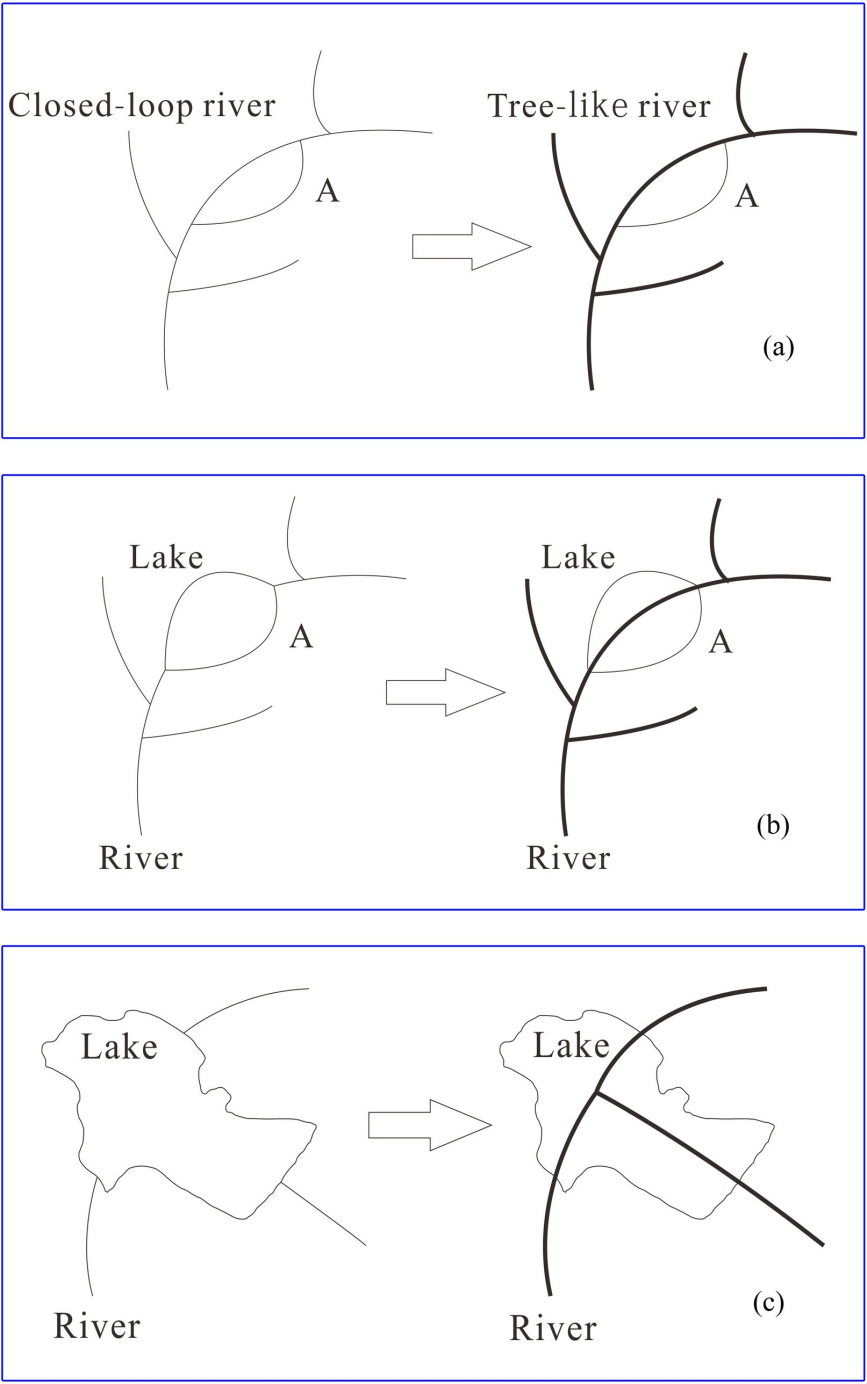
Transformation of complex dendritic river networks into simple dendritic river networks: (a) closed-loop river transformation; (b) river connected to the lakes transformation: case 1; and (c) rivers connected to the lakes transformation: case 2.

Although river networks are usually represented by a "tree" in graph theory, the establishment of a tree is often restricted by non-dendritic features, such as braided tributaries, closed loops, and interconnected lakes. Based on DTTs that contain semantic information, complex features in a river network, such as closed loops or lakes, can be identified in an intelligent manner. A method for transforming complex and diverse dendritic river networks into basic dendritic river networks was established as shown in Fig 2.

The processing of river networks that contain loops has been detailed in previous studies [29] and the methods proposed by Chithambaram *et al*. [30] and Haunert & Sester [31] may be used to perform the structured compensation of rivers connected to the lakes. Owing to the generalization of basic dendritic river networks is the core of complex dendritic river network generalizations, this paper mainly studies the development of a structured method of generalization for basic dendritic river networks.

### Constructing stroke connections to generate hierarchical relationships

The ‘stroke’ concept originates from the law of continuity in the Gestalt principles of perception, and this concept is derived from the drawing of a continuous line using a single brush stroke [32]. Compared with current methods, stroke-based selection methods provide an effective tool for simulating the visual principles of perception in manual selection.

#### Determining the starting nodes of stroke connections

The choices of starting and ending nodes have a decisive impact on differentiating the main stems and tributaries in river networks using stroke features. River sources and estuaries must be chosen before starting nodes can be identified. Almost all rivers have only one estuary in their lower reaches; therefore, stroke connections were constructed by tracing the estuaries to the sources via a downstream-to-upstream approach.

When problems related to data acquisition quality or river network development lead to multiple estuaries, investigative calculations must be performed for each possible estuary to trace a dendritic river network that is constrained by stroke characteristics (henceforth RivStroke). The node that connects more rivers is then selected as the estuary. If the numbers of rivers in each RivStroke for different nodes are equivalent, the lengths of RivStroke are then compared, and the node whose stroke has the longer length is selected as the estuary. This process determines the ending node that is the most appropriate estuary.

#### Basic principles of stroke connections

RivStrokes constrained by stroke features are generally created according to the following principles:

1. Semantic consistency. River arcs that have the same name will be prioritized for stroke connection. The combination of different types of river arcs (e.g., perennial rivers, dried rivers, and seasonal rivers) to form strokes is based on length priority and directional consistency as described below.
2. Length priority. The length of a reach is another important factor for creating stroke connections. At any fork in a river, the longer arc will be preferred for stroke connection.
3. Directional consistency. Connected arcs that conform to the law of continuity will have natural connection transitions and inter-arc angles approaching 180°. Thus, the suitability of a pair of connected arcs for stroke connection depends on how well they conform to these conditions.

#### Iterative stroke construction

Based on the aforementioned principles of stroke connection, constructing stroke connections in a dendritic river network is described in this section, and the main steps are as follows:

Step 1: The estuary is selected as the starting node for tracing the river. The arc that connects to the estuary is defined as the tracing arc. This process yields the other node of the tracing arc, which is used as the tracing node.
Step 2: The arcs connected to the tracing node compose the set of candidate arcs and the angles between these candidate arcs are also calculated.
Step 3: Stroke connections are constructed based on river name, length, and angle in descending priority. In addition, each arc can only be a part of one stroke. This process is repeated until the candidate set is empty, then the processing of one tracing node is completed.
Step 4: Continue to get tracking nodes based on the tracking arcs, and repeat the aforementioned procedure of constructing stroke connections until all nodes in the river network have been processed.

#### Hierarchical relationships

**Fig 3:**
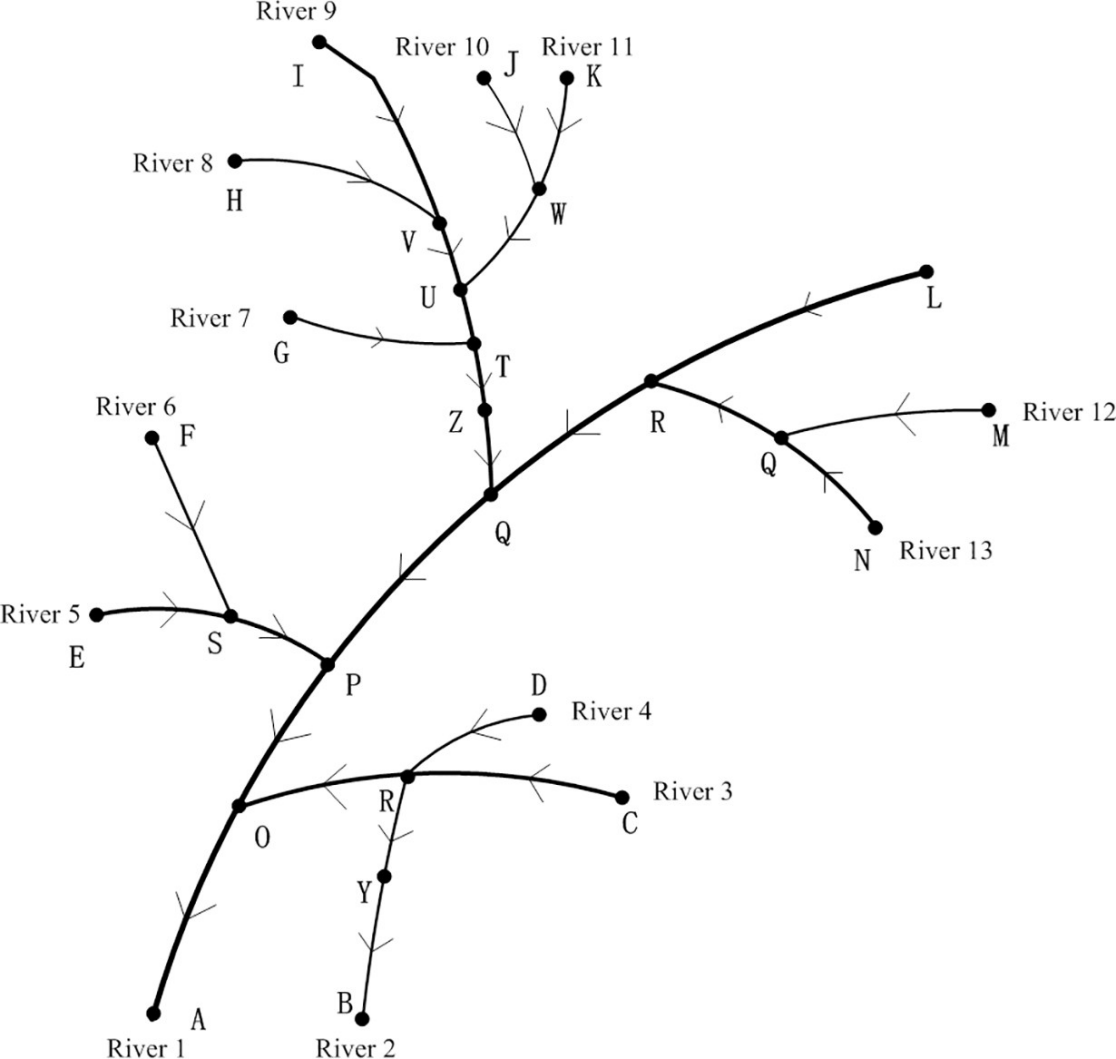
Hierarchical structure of a river network.

The hierarchical structure of the river network is hidden within the iterative process of constructing RivStrokes. All RivStrokes traced from the estuary are Level 1 rivers, whereas the RivStrokes traced from the confluences of Level 1 rivers are Level 2 rivers. The hierarchical relationships of each river in the network are obtained in this manner. A river that leads away from a bifurcation point of a river is treated as a branch of the river network, and the same level is assigned to all branches of a bifurcation point, as shown in Fig 3. It shows only one Level 1 river, River 1 (L1~L5); five Level 2 rivers, i.e., River 3 (L6~L9), River 5 (L11~L12), River 9 (L14, L15, L16, L17, L18), River 13 (L24, L25), and River 2 (L7, L8); seven Level 3 rivers, i.e., River 4 (L10), River 6 (L13), River 7 (L19), River 8 (L20), River 11 (L21, L22), and River 12 (L26); and only one Level 4 river, i.e., River 10 (L23).

### Automated selection based on hierarchical relationships

Several factors must be considered during the river selection processes, including river length, network density, network type, level. River hierarchies based on the constraints of stroke features are important for judging the importance of rivers because they provide a comprehensive account of a river’s semantics, geometry, and topology.

#### Determining the number of rivers for selection

The square-root-type model is used to determine the number of rivers for selection in this paper. The square-root-type model is an equation for surface feature selection that was proposed by German cartographer F. Topfer. This model is shown in Eq (1):

$$n_{F}=n_{A}\sqrt{(M_{A}/M_{F}{)}^{x}}$$ (1)

where *n*_*F*_ is the new number of objects, *n*_*A*_ is the original number of objects, *M*_*A*_ is the original scale denominator, *M*_*F*_ is the target scale denominator, and *x* is the empirical coefficient. The value of *x* is affected by river system density, the span between the original map and the target scales, etc., and generally ranges from 1 to 5.

#### Selection via hierarchical elimination from outside to inside

River selection can be conducted using “river preservation” or “river elimination” methods. This study proposes a process of hierarchical elimination “from outside to inside” based on the topological relationships of river networks. In the topological structure of a dendritic river network, river arcs may be divided into two types: “trunk arcs” and “dangling arcs”. Trunk arcs are central arcs that connect other arcs, usually the main stems of a river network. Dangling arcs refer to river arcs that have an end that does not connect to any other arcs, and they are often located at the margins of river networks, which are usually small river networks without any tributaries. After a river network is divided into levels, certain dangling arcs only connect to higher-level arcs, and their importance is one level lower than the arcs they feed into. Arcs of this type are known as the “sub-dangling arcs” of higher-level arcs.

River selection via hierarchical elimination is a procedure in which sub-dangling arcs are processed cyclically. Each cycle includes four basic steps.

Step 1: The entirety of the river network is traversed, and the number of sub-dangling arcs in each level is counted according to the river network’s hierarchy.
Step 2: The unequal allocation of selection numbers, in which number of arcs for elimination is allocated to each level, and the allocations are calculated using Eq (2):

$$n_{Ci}=n_{C}\times \frac{n_{mi}}{n_{m}}$$ (2)

where *n*_*Ci*_ is the number of rivers being eliminated at level *i*, *n*_*C*_ is the total number of rivers being eliminated, *n*_*mi*_ is the number of rivers in level *i*, and *n*_*m*_ is the total number of rivers across all levels. If a sub-watershed is only a small river network with few or no tributaries, then a river may not be eliminated. Therefore, if a watershed contains many small river networks, the river networks are added together and treated as a singular entity for the purposes of this calculation. The selected number of rivers is then rounded to an integer.

Step 3: The number of rivers for elimination from each level is allocated to each sub-watershed.
Step 4: In each sub-watershed, rivers are eliminated at each level based on the river length and river spacing.

This process is repeated until the total number of rivers that has been removed matches the total number of eliminations determined by the square-root-type model.

**Fig 4:**
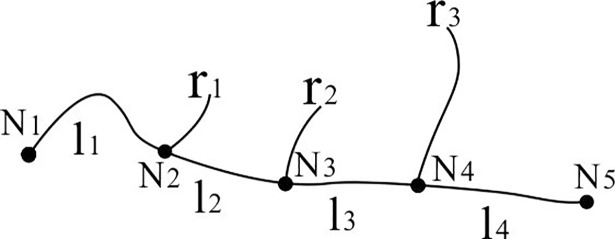
Method for calculating river spacing.

River selection processes must account for the spatial characteristics of the rivers. River length (L) and spacing (D) are the indices that are commonly used to maintain the spatial density of rivers. In practice, however, a clear method for distinguishing between long or short rivers or small or large river spacing still is not available. Therefore, Eq (3) was proposed for calculating the importance factor, *I*_*R*_, of each river:

$$I_{R}=\alpha L+\beta D$$ (3)

where *α* and *β* are the weighting coefficients for river length (*L*) and spacing (*D*), respectively. The values of these coefficients range from 0 to 1, and the sum of these values is 1. The coefficient values are also independent of the map scale and river network characteristics and may be determined using an “incremental method” if sufficient sample data points are available. In this work, the spacing of a river is defined as the sum of distances between the main stem confluence of the river and the main stem confluences of its “forward adjacent” and “backward adjacent” rivers. In Fig 4, the river spacing of r_2_, which has adjacent river flows on both of its sides, is the sum of l_2_ (the distance between N_2_ and N_3_) and l_3_ (the distance between N_3_ and N_4_). For a river similar to r_1_ that only has an adjacent river on one of its sides, its river spacing is the sum of l_1_ (the distance between N_2_ and N_1_) and l_2_ (the distance between N_2_ and N_3_). When two rivers have the same importance factor (*I*_*R*_), selection is then performed according to the length index. If their length indices are also the same, i.e., the two rivers have the same length and spacing and the same weighting coefficients, then one of the rivers will be selected at random.

#### Determination of α and β

The calculations of the weighting coefficients for river length (L) and spacing (D), α and β, are crucial aspects of our method. These weighting coefficients are decisive factors in the ultimate results of the selection. Here, an “incremental method” [33, 34] was used to calculate the values of α and β using sample data.

**Fig 5:**
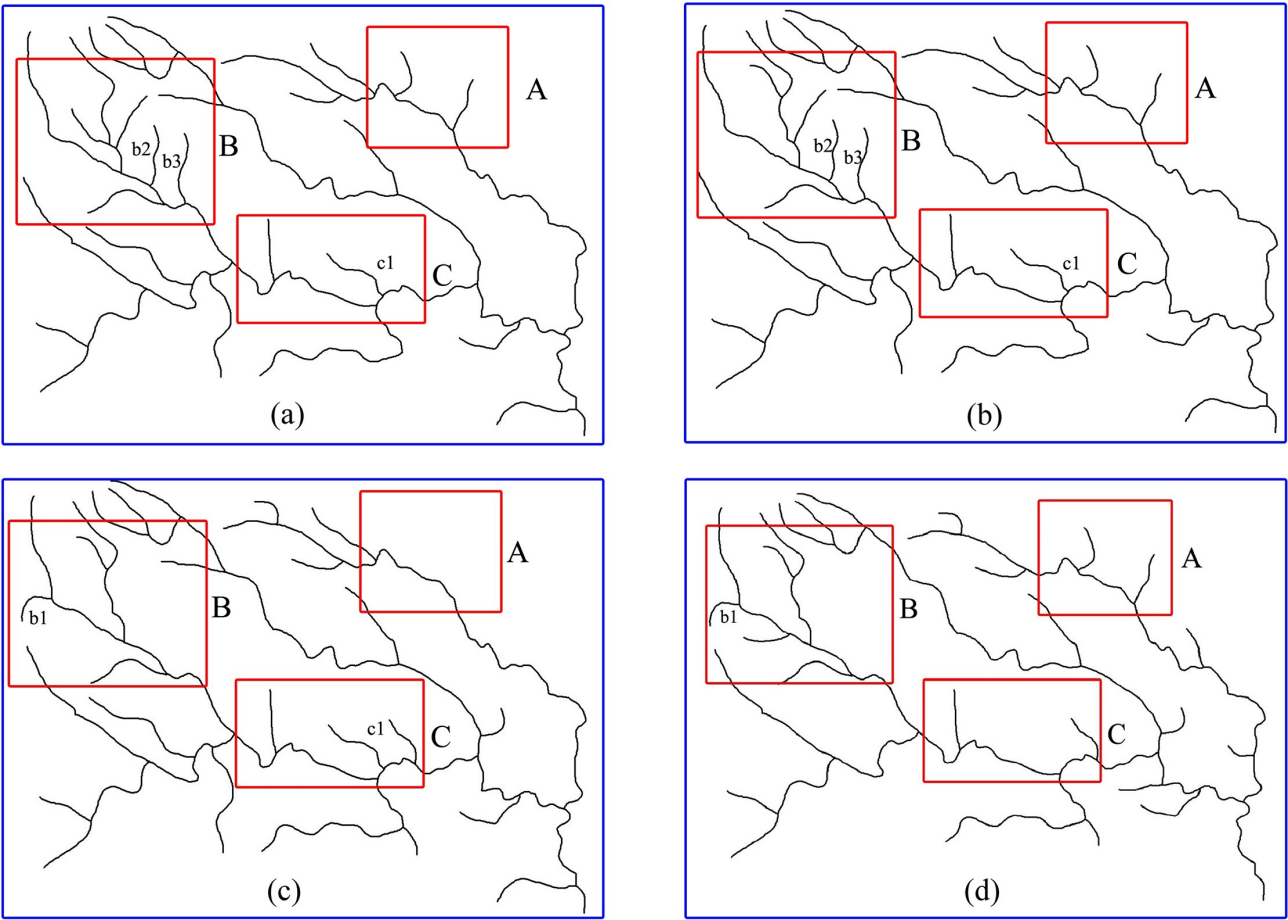
Example values of *α* and *β* and the corresponding results from the selection process.

Sample data were obtained from He [10], and these data constitute a 1:200000 map of a basin in the western region of Hubei Province that contains 68 rivers (Fig 5). This map was generalized to 1:500000 to demonstrate how *α* and *β* are determined. Increments of 0.1 were used for *α* and *β*, thus producing 11 sets of data between 0 and 1. These data sets were used to perform the selection process. Experimental results are shown in Fig 5. When *α* > *β*, the arcs that were selected in experimental areas B and C are rivers with long lengths (Fig 5A and 5B). In contrast, Fig 5C and 5D shows that the distribution of rivers in this river network is denser, and the river spacing is smaller. As *α* decreases and *β* increases, some of the shorter arcs that have wider river spacing were retained, such as b1 in Fig 5C and 5D. In addition, arcs that are long but have short river spacing were eliminated, such as b2 and b3 in Fig 5A and 5B and c1 in Fig 5C and 5D. A comparison between the results of this selection with the manually simplified map from He [10]—shows that the weighting coefficient values that produce the most similar results to He [10] are *α* = 0.8 and *β* = 0.2 (Fig 5B).

### Experiments: Reliability and validity of the proposed method

This paper presents a universal selection method for dendritic river network generalization. In order to verify the accuracy and universality of proposed method, a small sample dataset and a large actual dataset were used in the experiment. The experiment was conducted by using the WJ-III mapping workstation developed by the Chinese Academy of Surveying and Mapping, which provided geometric computation, topology construction, spatial relationship and other class libraries in the C++ platform.

#### Accuracy experiment

**Fig 6:**
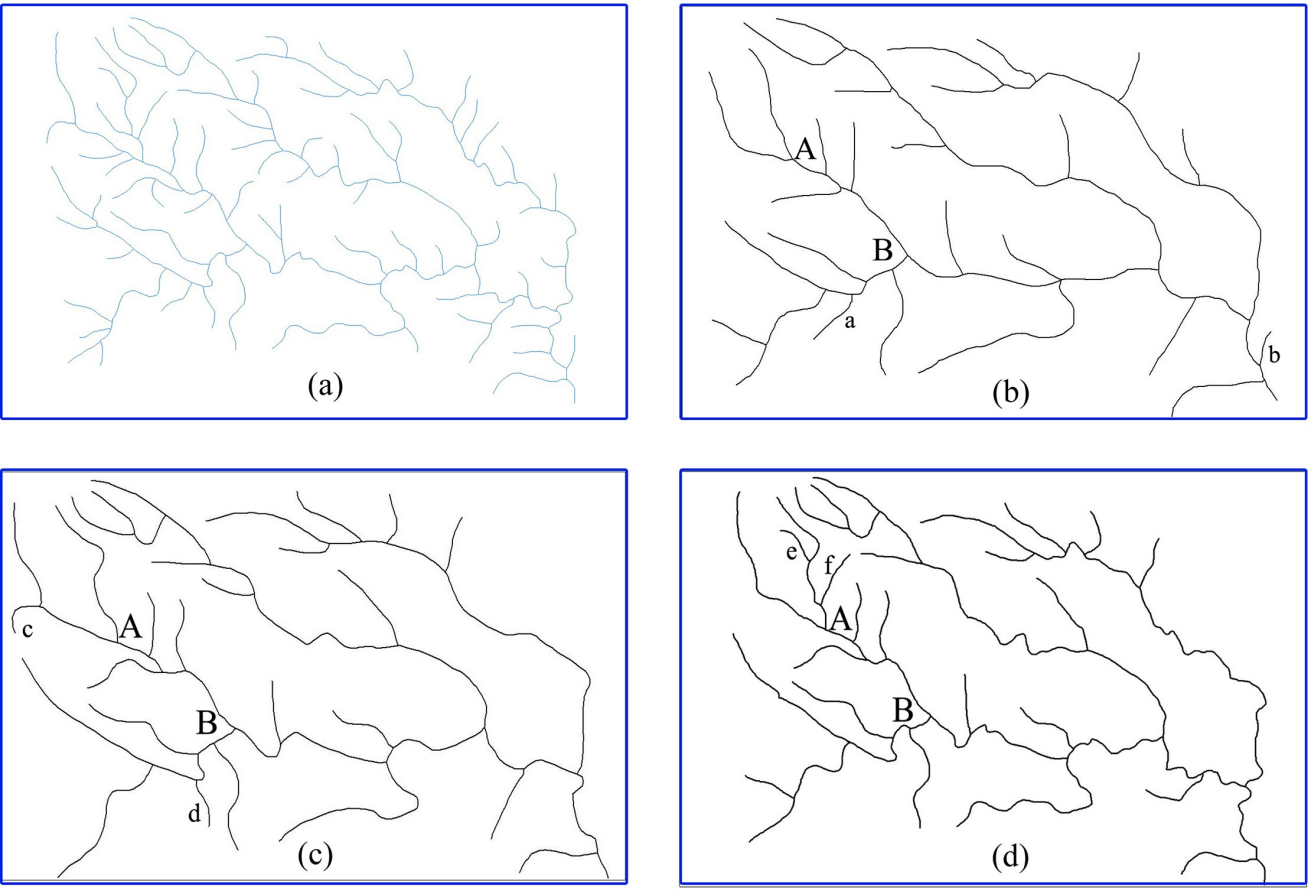
Comparison diagrams for the experiment on the accuracy of our method: (a) 1:200000 original data, (b) a 1:500000 simplified map generated via manual selection method, (c) a 1:500000 un-simplified map generated via hierarchical preservation selection method [35], and (d) a 1:500000 un-simplified map generated via our method.

To verify the accuracy of the method proposed in this paper, the same 1:200000 basin map of a western region in Hubei Province from He [10] (Fig 6A) was used as sample data. The target scale for the selection process was 1:500000. A comparison was then conducted between manual selection method (Fig 6B), which was already simplified, and the hierarchical preservation selection method described in Zhang [35] (Fig 6C). Visual comparisons were performed to evaluate the results of river selection.

#### Universality experiment

The universality of the method proposed in this study was tested using a 1:10000 actual dataset in Hubei Province produced by the National Geographic Conditions Survey. The area of the experimental region is 90.91×106.56 km^2^, and this region contains a well-developed basin with 944 rivers. During the preprocessing of the data, closed loops in the river network were removed to make it a fully dendritic structure. The estuary reaches were then identified, and a “bottom to top” approach was used to iteratively construct a hierarchical river network constrained by stroke features.

Three target scales (1:50000, 1:100000 and 1:250000) were selected, and the square-root rule was used to calculate the total number of rivers for selection. The value of *x* were set to 1, 1, and 2 for the three target scales. The *α* and *β* values were 0.8 and 0.2, respectively. Finally, the method proposed in this study was used to select rivers, without performing river simplification.

## Results

### Results of accuracy experiment

The experimental parameters were as follows: x was set to 2 in Eq (1), which is consistent with the value used in Zhang [35], while the values of α and β were 0.8 and 0.2, respectively. A 1:500000 basin map (Fig 6D), which has not been simplified, was subsequently obtained using our method of selection.

The selection results via our method in Fig 6D were high consistency with the results via manual selection method in Fig 6B and via hierarchical preservation selection method in Fig 6C. As shown in Fig 6D, regional differences in river density and the spatial structure of the river network are well preserved and the selected rivers maintain a good balance between river length and spacing, the short and closely spaced rivers are not observed on the map after generalization. The results prove that the new method is reliable, with good accuracy.

### Results of universality experiment

**Fig 7:**
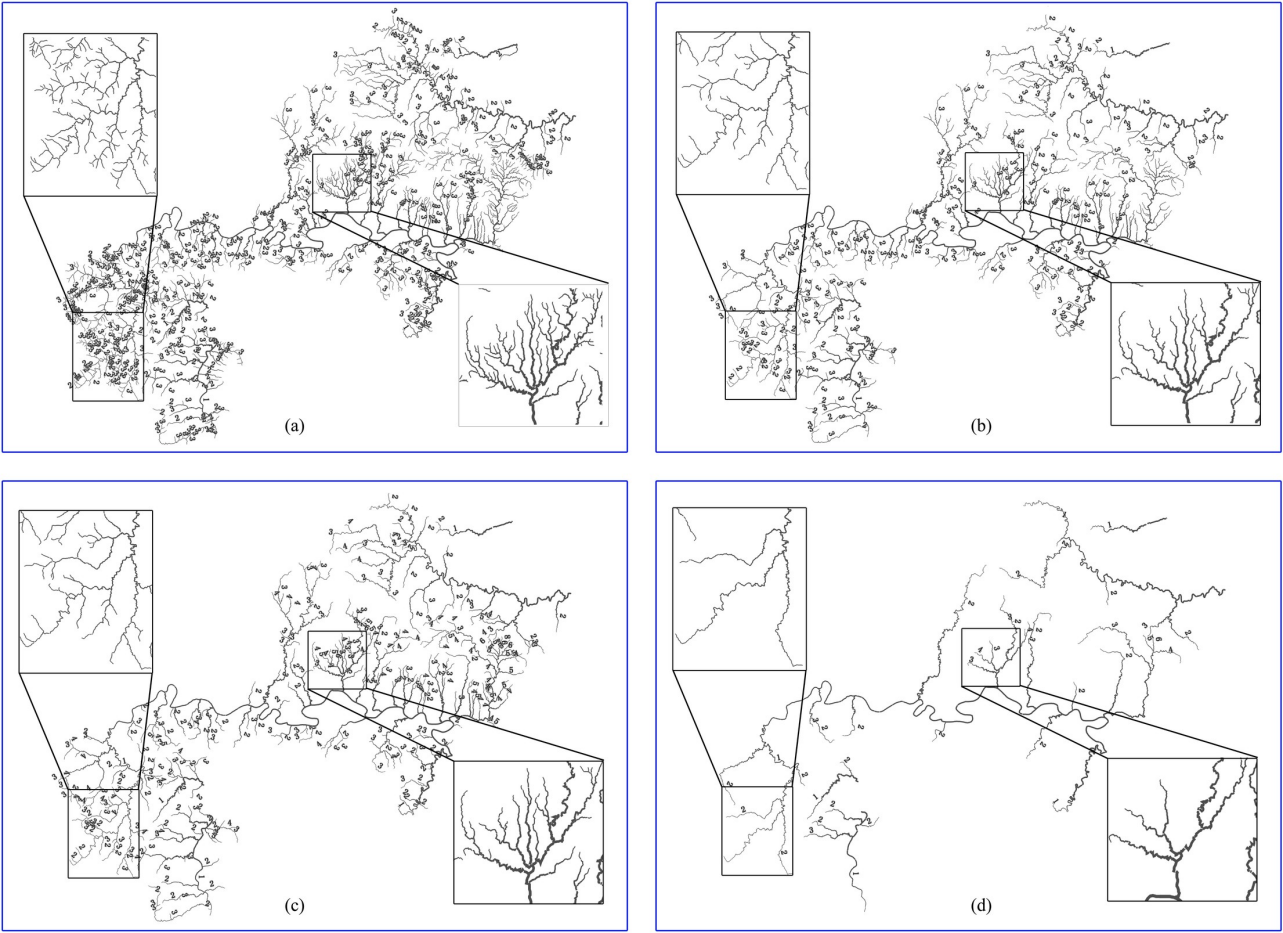
Results of selections at different target scales based on actual river dataset: (a) the 1:10000 original map, (b) a 1:50000, (c) a 1:100000, and (d) a 1:250000 selection results.

The results of the selections at each target scale are shown in Fig 7B and 7D, whereas the number of rivers selected for each level is shown in Table 1.

**Table 1:** The number of rivers selected for each level at different target scales.

Scale	x	Number of rivers	Level 1	Level 2	Level 3	Level 4	Level 5	Level 6	Level 7	Level 8	Level 9
**1:10000**	1	944	6	203	351	245	100	27	9	2	1
**1:50000**	1	418	6	111	154	100	35	9	2	1	0
**1:100000**	1	296	6	90	107	66	20	5	1	1	0
**1:250000**	2	40	6	21	8	3	1	1	0	0	0

Fig 7 shows that the application of the methods presented in this paper results in an accurate reflection of the spatial distribution characteristics of the river network and differences in density between the sub-watersheds among the various target scales. This method can effectively avoid the problems of elimination of entire sub-watersheds due to the elimination of the higher-level rivers, and the connectivity of the river network’s margins maintained. Moreover, no disconnections in the river network’s margins were observed.

Table 1 clearly shows that the “outside to inside” approach for hierarchical elimination and selection has effectively preserved the trunk of the river network. Level 1 rivers were always preserved due to their central locations in the inner layers. The selection of each tributary maintained the proportion of tributaries in the river network, thereby preserving the characteristics of the spatial distribution of the river network. Furthermore, as the target scale decreased, the river networks with lower levels became increasingly prioritized for elimination. The results indicate that our method can be applied to the selection of multi-scale dendritic river network generalization.

Table 2 shows the computational time required for automatic selection of river network from 1:10000 to three different target scales. The time spent in this three execution process is basically the same, which was nearly 4s. Time is mainly spent in the process of constructing stroke connections to generate hierarchical and the automated selection based on hierarchical relationships.

**Table 2:** The computational time for different target scales.

Original scale	1:10000		
target scales	1:50000	1:100000	1:250000
time (s)	4.087	4.103	4.119

## Conclusions

The structured selection of rivers is a key and difficult problem in dendritic river network generalization. Therefore, this paper proposed a hierarchical elimination selection method for generalizing dendritic river networks via a combination of semantic, geometric, and topological characteristics of river networks with the Gestalt perception principles. It can capture better preservation of the original spatial distribution characteristics of dendritic river networks. Through the experiments, the main conclusions are drawn as follows:

1. The selection results of the presented method are high consistency with those obtained by manual selection method and the method in Zhang [35] with the same sample dataset, which proves that the presented method is of good accuracy.
2. The selection results of actual dataset (include nearly 1000 river networks) for multi-scale generalization well maintained the spatial distribution characteristics of the river network and represent differences in density between the sub-watersheds among the various target scales, which verifies that the presented method is of good universality.
3. The automatic selection time of our method is about 4s for multi-scale generalization with the actual dataset based on the WJ-III mapping workstation in C++ platform, which indicates that the presented method is of high computational efficiency.

This study addressed the hierarchical elimination selection of dendritic river networks; however, it did not fit the dendritic river network with closed loops, and except for the scale between 10000 and 250000, the validity of this method has not been confirmed. In addition, the values of α and β were determined empirically. Therefore, the constraints imposed by residential areas and other factors on river selection and the applicability of our method to other types of river networks (e.g., trellis, irregular drainage networks, etc.) will be investigated in future studies.
